# Increased risk of secondary lung cancer in patients with tuberculosis: A nationwide, population-based cohort study

**DOI:** 10.1371/journal.pone.0250531

**Published:** 2021-05-07

**Authors:** Li-Ju Ho, Hung-Yi Yang, Chi-Hsiang Chung, Wei-Chin Chang, Sung-Sen Yang, Chien-An Sun, Wu-Chien Chien, Ruei-Yu Su

**Affiliations:** 1 Division of Endocrinology and Metabolism, Department of Internal Medicine, Tri-Service General Hospital, National Defense Medical Center, Taipei, Taiwan; 2 Division of Clinical Pathology, Department of Pathology, Tri-Service General Hospital, National Defense Medical Center, Taipei, Taiwan; 3 School of Public Health, National Defense Medical Center, Taipei, Taiwan; 4 Department of Oral and Maxillofacial Surgery, Tri-Service General Hospital, National Defense Medical Center, Taipei, Taiwan; 5 Department of Medical Research, Tri-Service General Hospital, National Defense Medical Center, Taipei, Taiwan; 6 Graduate Institute of Medical Sciences, National Defense Medical Center, Taipei, Taiwan; 7 Department of Public Health, College of Medicine, Fu-Jen Catholic University, New Taipei City, Taiwan; 8 Big Data Research Center, College of Medicine, Fu-Jen Catholic University, New Taipei City, Taiwan; 9 Graduate Institute of Life Sciences, National Defense Medical Center, Taipei, Taiwan; 10 Department of Pathology and Laboratory Medicine, Taoyuan Armed Forces General Hospital, Taoyuan, Taiwan; Universiti Putra Malaysia, MALAYSIA

## Abstract

**Background:**

Tuberculosis (TB) presents a global threat in the world and the lung is the frequent site of metastatic focus. A previous study demonstrated that TB might increase primary lung cancer risk by two-fold for more than 20 years after the TB diagnosis. However, no large-scale study has evaluated the risk of TB and secondary lung cancer. Thus, we evaluated the risk of secondary lung cancer in patients with or without tuberculosis (TB) using a nationwide population-based dataset.

**Methods:**

In a cohort study of 1,936,512 individuals, we selected 6934 patients among patients with primary cancer and TB infection, based on the International Classification of Disease (ICD-p-CM) codes 010–011 from 2000 to 2015. The control cohort comprised 13,868 randomly selected, propensity-matched patients (by age, gender, and index date) without TB exposure. Using this adjusted date, a possible association between TB and the risk of developing secondary lung cancer was estimated using a Cox proportional hazards regression model.

**Results:**

During the follow-up period, secondary lung cancer was diagnosed in 761 (10.97%) patients with TB and 1263 (9.11%) patients without TB. After adjusting for covariates, the risk of secondary lung cancer was 1.67 times greater among primary cancer in the cohort with TB than in the cohort without TB. Stratification revealed that every comorbidity (including diabetes, hypertension, cirrhosis, congestive heart failure, cardiovascular accident, chronic kidney disease, chronic obstructive pulmonary disease) significantly increased the risk of secondary lung cancer when comparing the TB cohort with the non-TB cohort. Moreover, the primary cancer types (including head and neck, colorectal cancer, soft tissue sarcoma, breast, kidney, and thyroid cancer) had a more significant risk of becoming secondary lung cancer.

**Conclusion:**

A significant association exists between TB and the subsequent risk for metastasis among primary cancers and comorbidities. Therefore, TB patients should be evaluated for the subsequent risk of secondary lung cancer.

## Introduction

Tuberculosis (TB) presents a global threat in both developing and developed countries. TB is caused by bacteria (*Mycobacterium tuberculosis*) and most often affects the lungs. According to the World Health Organization, 10 million people become ill with TB annually. Despite being a preventable and curable disease, 1.5 million people die from TB each year–making it the world’s top infectious killer [1].

The lung is the frequent site of metastatic focus. About 20% to 54% of malignant tumors developing elsewhere in our body have pulmonary metastasis [2, 3]. This is so-called secondary lung cancer when cancer cells have spread to the lungs from cancer that started elsewhere in the body. It is also called metastatic cancer to the lungs and differs from the definition of primary lung cancer that has originated in the lungs. A previous study demonstrated that TB might increase primary lung cancer risk by two-fold for more than 20 years after the TB diagnosis [4]. However, no large-scale study has evaluated the risk of TB and secondary lung cancer. Thus, a nationwide, population-based, matched cohort study is needed to clarify the association between TB infection and secondary lung cancer. Furthermore, we have conducted this study to investigate whether comorbidities could attenuate the risk of developing secondary lung cancer after TB infection.

## Material and methods

### Data source and ethics statement

The National Health Insurance (NHI) program began in Taiwan in 1995 and covers more than 99% of the entire population (or more than 23 million people). The data for this study were collected from the NHI Research Database (NHIRD) of Taiwan, which uses the International Classification of Diseases, 9^th^ Revision, Clinical Modification (ICD-9-CM) codes to record diagnoses. Therefore, we used the NHIRD inpatient and outpatient databases and the Registry of Beneficiaries. Patient confidentiality was ensured by double-encrypted identifiers in the NHIRD. The Institutional Review Board of Tri-Service General Hospital approved this study (TSGHIRB No.B-109-44), and the committee waived the need for written informed consent.

### Study design and population

**Fig 1:**
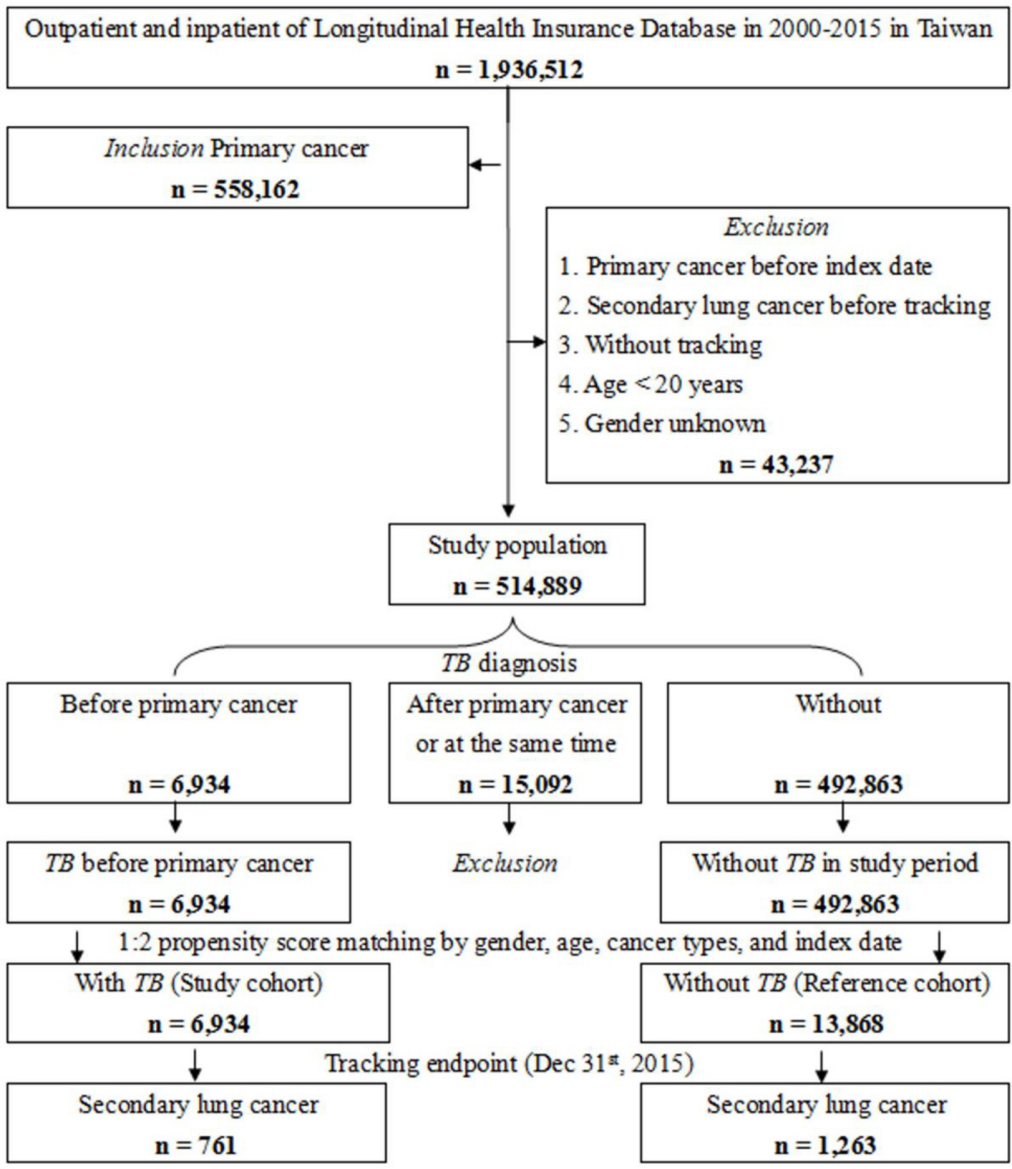
Flowchart of study sample selection.

The study design and specific patient characteristics, including inclusion and exclusion criteria, are shown in Fig 1. The control cohort (non-TB patients) was randomly matched with TB patients according to age, sex, and index date (two controls for each TB patient) using the same exclusion criteria. The study cohort included 514,889 patients aged ≥20 years who had been diagnosed with cancer except for lung origin (ICD-9-CM codes in S1 Table) from 2000 to 2015. The index date was designated as the first clinical visit for primary cancer. The exclusion criteria were: diagnosis with primary cancer before 2000, secondary lung cancer before tracking, patients without tracking, age <20 years, and unknown gender. The ratio of primary cancer patients with TB to patients without TB in the study period was maintained at 1:2 to enhance the power of the statistical tests employed, particularly regarding the stratification analysis. Using these criteria, 6934 patients with TB infection and 13,868 patients without TB infection were identified.

### Covariates

We examined the sociodemographic factors in the case and control groups, such as age, monthly income, comorbidity, urbanization level, and hospital level. The patients were divided into three groups: 20–44 years, 45–69 years, and ≥70 years. Their monthly income in New Taiwan Dollars was divided into three groups: <18,000, 18,000–34,999, and ≥35,000. Seven comorbidities (ICD codes as in S1 Table), such as diabetes (DM)、hypertension (HTN)、cirrhosis、congestive heart failure (CHF)、cardiovascular accident (CVA)、chronic kidney disease (CKD)、and chronic obstructive pulmonary disease (COPD), were also considered into our study (using ICD9 codes in S1 Table). The patients were categorized into four urbanization levels. The three hospital levels where patients sought medical attention were also considered: medical centers, regional hospitals, and local hospitals.

### Study outcome

All study participants were followed from the index date until the onset of secondary lung cancer (ICD-9-CM codes: 197.0), withdrawal from the NHI program, or the end of 2015. The covariates included were included those mentioned previously.

### Statistical analysis

We performed all analyses using SPSS software version 22 (SPSS Inc., Chicago, Illinois, USA). χ2 and t-tests were used to evaluate the distribution of categorical and continuous variables, respectively. Fisher’s exact test was used for categorical variables to examine the statistical differences between the two cohorts. The multivariate Cox proportional hazards regression analysis was used to determine the risk of secondary lung cancer. The results were presented as a hazard ratio with a 95% confidence interval (CI). The difference in the risk of secondary lung cancer between TB-infected subjects and control groups was estimated using the Kaplan-Meier method with the log-rank test. A two-tailed *p*-value < .05 was considered statistically significant.

From 2000 to 2015, a total of 558,162 patients with primary cancer were enrolled in this study in accordance with our inclusion criteria. Secondary lung cancer was observed in 761 of 6934 TB-infected patients, and in 1263 of 13,868 non-TB-infected patients. Table 1 lists demographic characteristics and comorbidities of the TB (6934) and non-TB cohorts (13,868) during this time. In both cohorts, approximately 90% were older than 45 years of age, 72% were male, and the proportion by age and sex were similar. All comorbidities, except CVA, were significantly different in the TB cohort than in the endpoint non-TB cohort: DM (17.41% *vs* 13.36%; *p* < .001), HTN (16.33% *vs* 18.73%; *p* < .001), cirrhosis (4.12% *vs* 2.40%; *p* < .001), CHF (3.87% *vs* 2.63%; *p* < .001), CKD (8.48% *vs* 5.70%; *p* < .001), and COPD (11.52% *vs* 4.25%; *p* < .001). The TB cohorts had a higher proportion of individuals living at the lowest urbanization level city (19.12% *vs* 14.08%; *p* < .001) and call for treatment in local hospital (15.10% *vs* 11.85%; *p* < .001).

**Table 1:** Demographic characteristics and comorbidities of study participants.

*TB*	Total		With		Without		*P*
Variables	n	%	n	%	n	%	
**Overall**	20,802		6934	33.33	13,868	66.67	
**Secondary lung cancer**							< .001
Without	18,778	90.27	6173	89.03	12,605	90.89	
With	2024	9.73	761	10.97	1263	9.11	
**Gender**							.999
Male	15,069	72.44	5023	72.44	10,046	72.44	
Female	5733	27.56	1911	27.56	3,822	27.56	
**Age (yrs)**	67.50 ± 14.19		67.05 ± 13.63		67.73 ± 14.46		< .001
**Age groups (yrs)**							< .001
20–44	1713	8.23	473	6.82	1240	8.94	
45–69	9577	46.04	3125	45.07	6452	46.52	
≧70	9512	45.73	3336	48.11	6,176	44.53	
**Insurance premium (NT$)**							.936
<18,000	20,450	98.31	6818	98.33	13,632	98.30	
18,000–34,999	291	1.40	97	1.40	194	1.40	
≧35,000	61	0.29	19	0.27	42	0.30	
**DM**							< .001
Without	17,742	85.29	5727	82.59	12,015	86.64	
With	3060	14.71	1207	17.41	1853	13.36	
**HTN**							< .001
Without	17,072	82.07	5802	83.67	11,270	81.27	
With	3730	17.93	1132	16.33	2598	18.73	
**Cirrhosis**							< .001
Without	20,183	97.02	6648	95.88	13,535	97.60	
With	619	2.98	286	4.12	333	2.40	
**CHF**							< .001
Without	20,169	96.96	6666	96.13	13,503	97.37	
With	633	3.04	268	3.87	365	2.63	
**CVA**							.629
Without	19,807	95.22	6608	95.30	13,199	95.18	
With	995	4.78	326	4.70	669	4.82	
**CKD**							< .001
Without	19,423	93.37	6346	91.52	13,077	94.30	
With	1379	6.63	588	8.48	791	5.70	
**COPD**							< .001
Without	19,413	93.32	6135	88.48	13,278	95.75	
With	1389	6.68	799	11.52	590	4.25	
**Urbanization level**							< .001
1 (The highest)	7125	34.25	2183	31.48	4942	35.64	
2	9382	45.10	3067	44.23	6315	45.54	
3	1017	4.89	358	5.16	659	4.75	
4 (The lowest)	3278	15.76	1326	19.12	1952	14.08	
**Level of care**							< .001
Hospital center	9793	47.08	2898	41.79	6895	49.72	
Regional hospital	8318	39.99	2989	43.11	5329	38.43	
Local hospital	2691	12.94	1047	15.10	1,644	11.85	

*P*: Chi-square / Fisher’s exact test for categorical variables and t-test for continuous variables

### Secondary lung cancer incidence and risk

Table 2 presents factors of secondary lung cancer using Cox regression and Fine & Gray’s competing risk model. According to our study, the risk of secondary lung cancer was 1.671 times greater in the TB cohort than in the non-TB cohort (aHR = 1.671; 95% CI = 1.525–1.832; *p* < .001) after adjusting for gender, age, insurance premium, related comorbidities, urbanization level, and level of care. All comorbidities were significantly higher in the TB cohort than in the non-TB cohort: DM (aHR = 1.472; 95% CI = 1.271–1.705; *p* < .001), HTN (aHR = 2.318; 95% CI = 1.993–2.696; *p* < .001), cirrhosis (aHR = 1.334; 95% CI = 1.017–1.750; *p* = .038), CHF (aHR = 3.017; 95% CI = 1.979–4.600; *p* < .001), CVA (aHR = 3.866; 95% CI = 2.676–5.584; *p* < .001), CKD (aHR = 2.562; 95% CI = 1.990–3.298; *p* < .001), and COPD (aHR = 2.238; 95% CI = 1.770–2.830; *p* < .001). Compared with the local hospital, the risk of secondary lung cancer was higher in the medical center (aHR = 2.332; 95% CI = 1.926–2.823; *p* < .001) and in the regional hospital (aHR = 1.728; 95% CI = 1.443–2.070; *p* < .001).

**Table 2:** Factors of secondary lung cancer using Cox regression and Fine & Gray’s competing risk model.

Model	Competing risk in the model							
Variables	Crude HR	95% CI	95% CI	*P*	Adjusted HR	95% CI	95% CI	*P*
***TB***								
Without	Reference				Reference			
With	1.478	1.350	1.618	< .001	1.671	1.525	1.832	< .001
**Gender**								
Male	1.083	0.983	1.194	.108	1.088	0.986	1.201	.093
Female	Reference				Reference			
**Age groups (yrs)**								
20–44	Reference				Reference			
45–69	1.004	0.664	1.124	.074	1.088	0.986	1.201	.093
≧70	1.018	0.865	1.197	.833	1.109	0.942	1.306	.215
**Insured premium (NT$)**								
<18,000	Reference				Reference			
18,000–34,999	1.096	0.528	1.201	.278	1.073	0.512	1.166	.220
≧35,000	1.512	0.755	3.027	.244	1.352	0.674	2.712	.396
**DM**								
Without	Reference				Reference			
With	1.849	1.601	2.136	< .001	1.472	1.271	1.705	< .001
**HTN**								
Without	Reference				Reference			
With	2.638	2.277	3.057	< .001	2.318	1.993	2.696	< .001
**Cirrhosis**								
Without	Reference				Reference			
With	1.127	0.860	1.478	.385	1.334	1.017	1.750	.038
**CHF**								
Without	Reference				Reference			
With	3.533	2.320	5.380	< .001	3.017	1.979	4.600	< .001
**CVA**								
Without	Reference				Reference			
With	4.848	3.360	6.995	< .001	3.866	2.676	5.584	< .001
**CKD**								
Without	Reference				Reference			
With	2.463	1.915	3.168	< .001	2.562	1.990	3.298	< .001
**COPD**								
Without	Reference				Reference			
With	2.249	1.786	2.833	< .001	2.238	1.770	2.830	< .001
**Urbanization level**								
1 (The highest)	0.779	0.600	1.011	.061	0.797	0.614	1.034	.088
2	0.859	0.702	1.439	.091	0.881	0.749	1.037	.127
3	0.938	0.779	1.474	.300	0.957	0.826	1.109	.559
4 (The lowest)	Reference				Reference			
**Level of care**								
Hospital center	2.465	2.071	2.933	< .001	2.332	1.926	2.823	< .001
Regional hospital	1.814	1.518	2.167	< .001	1.728	1.443	2.070	< .001
Local hospital	Reference				Reference			

HR = Hazard Ratio, CI = Confidence Interval, Adjusted HR: Adjusted variables listed in the Table

*P*: Chi-square/Fisher’s exact test for categorical variables and t-test for continuous variables

CI: Confidence Interval

**Fig 2:**
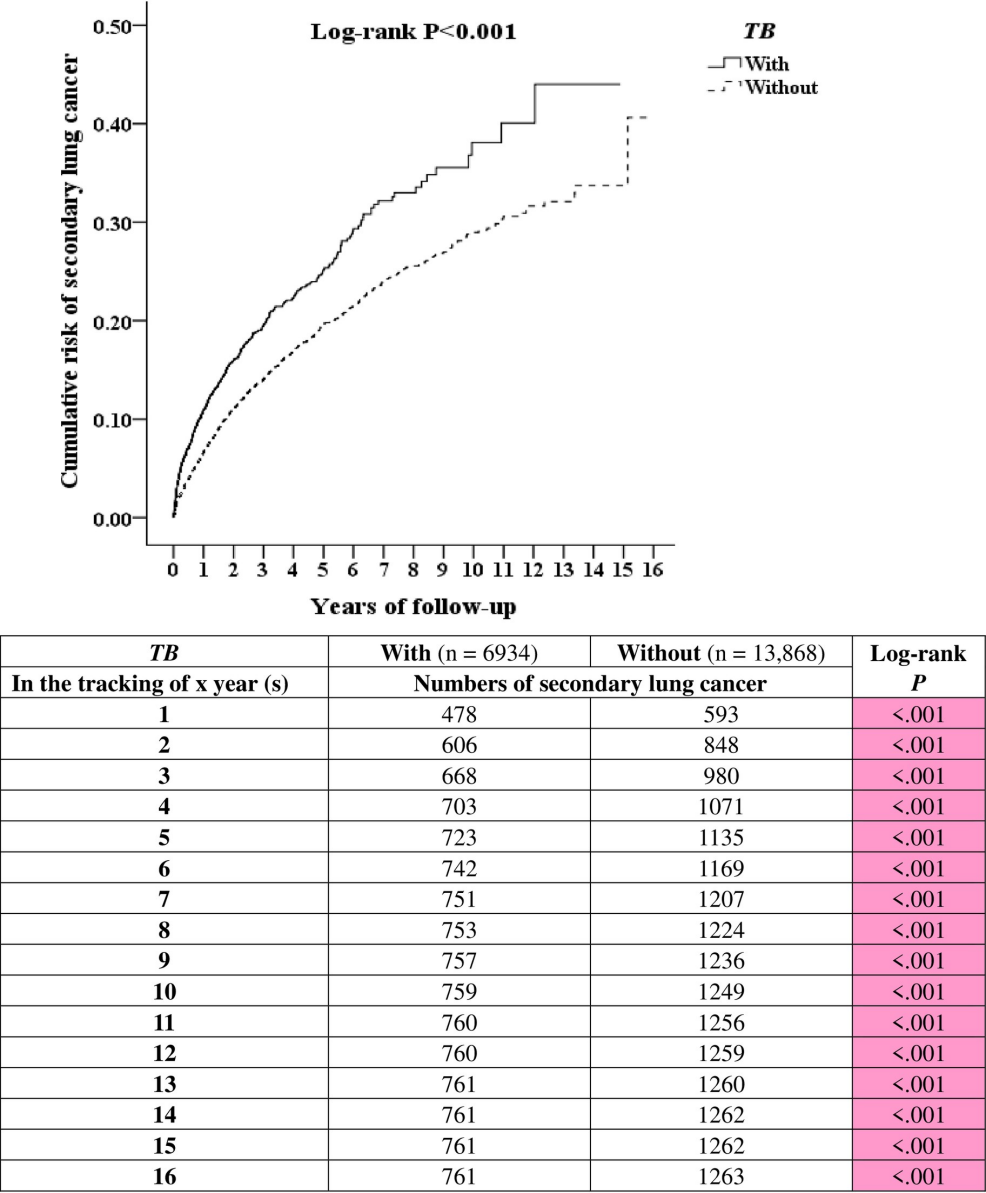
Kaplan-Meier for cumulative risk of secondary lung cancer among primary cancer patients aged 20 and over stratified by *TB* with the log-rank test.

Fig 2 compares the Kaplan-Meier curves for the cumulative incidence of secondary lung cancer between the TB and non-TB cohorts after 16 years of follow-up. The 1-, 5-, 11-, and 15-year actuarial rates of secondary lung cancer were 6.89%, 10.42%, 10.96%, and 10.97% in the TB cohort and 4.27%, 8.18%, 9.05%, and 9.10% in the non-TB cohort, respectively. This geography revealed that TB-infected patients had a significantly higher risk of developing secondary lung cancer than non-TB patients among primary cancer patients, even in the first year of tracking.

We found that 761 (10.97%) TB cohort members progressed to secondary lung cancer with 57,340 person-years of follow-up over 16 years, for an incidence rate of 1327 per 100,000 person-years. Conversely, only 1263 (9.10%) of the non-TB cohort members progressed to secondary lung cancer over the 124,884 person-years of follow-up for 16 years, for an incidence rate of 1011 per 100,000 person-years. Therefore, the incidence rate of osteoporosis was 1.671-fold higher in the TB cohort than in the non-TB cohort.

Table 3 shows the factors of secondary lung stratified by the variables listed using Cox regression and Fine & Gray’s competing risk model. All the factors show a significantly higher risk in TB-infected patients at every stratified level than in non-TB-infected patients.

**Table 3:** Factors of secondary lung cancer stratified by variables listed using Cox regression and Fine & Gray’s competing risk model.

*TB*	With			Without *(Reference)*			Competing risk in the model			
Stratified	Events	PYs	Rate (per 10^5^ PYs)	Events	PYs	Rate (per 10^5^ PYs)	Adjusted HR	95% CI	95% CI	*P*
**Overall**	761	57,340.90	1327.15	1263	124,884.91	1011.33	1.671	1.525	1.832	< .001
**Gender**										
Male	537	39,444.69	1361.40	908	89,000.07	1020.22	1.699	1.550	1.863	< .001
Female	224	17,896.21	1251.66	355	35,884.84	989.28	1.611	1.470	1.766	< .001
**Age group (yrs)**										
20–44	57	3026.89	1883.12	134	8816.02	1519.96	1.578	1.439	1.729	< .001
45–69	314	22,904.34	1370.92	559	52,083.65	1073.27	1.627	1.484	1.783	< .001
≧70	390	31,409.67	1241.66	570	63,985.24	890.83	1.775	1.619	1.946	< .001
**Insured premium (NT$)**										
<18,000	746	56,337.98	1324.15	1247	122,847.46	1015.08	1.661	1.516	1.821	< .001
18,000–34,999	11	865.00	1271.67	12	1715.19	699.63	2.315	2.112	2.537	< .001
≧35,000	4	137.92	2900.30	4	322.26	1241.22	2.976	2.715	3.262	< .001
**DM**										
Without	663	45,624.00	1453.18	1,144	104,122.51	1098.71	1.684	1.537	1.846	< .001
With	98	11,716.90	836.40	119	20,762.40	573.15	1.858	1.695	2.037	< .001
**HTN**										
Without	687	44,820.23	1532.79	1,139	96,117.53	1185.01	1.647	1.503	1.806	< .001
With	74	12,520.67	591.02	124	28,767.38	431.04	1.746	1.593	1.914	< .001
**Cirrhosis**										
Without	731	55,081.69	1327.12	1,239	121,468.64	1020.02	1.657	1.512	1.816	< .001
With	30	2259.21	1327.90	24	3416.27	702.52	2.407	2.196	2.638	< .001
**CHF**										
Without	748	54,419.08	1374.52	1,254	119,772.93	1046.98	1.672	1.525	1.833	< .001
With	13	2921.82	444.93	9	5111.98	176.06	3.218	2.936	3.528	< .001
**CVA**										
Without	746	53,561.46	1392.79	1244	115,641.45	1075.74	1.649	1.504	1.807	< .001
With	15	3779.44	396.88	19	9243.46	205.55	2.459	2.243	2.695	< .001
**CKD**										
Without	723	51,376.75	1407.25	1228	115,637.41	1061.94	1.688	1.540	1.850	< .001
With	38	5964.16	637.14	35	9247.50	378.48	2.144	1.956	2.350	< .001
**COPD**										
Without	711	49,078.54	1448.70	1238	117,246.40	1055.90	1.747	1.594	1.915	< .001
With	50	8262.37	605.15	25	7638.51	327.29	2.355	2.148	2.581	< .001
**Pneumoconiosis**										
Without	759	56,747.49	1337.50	1,263	124,776.18	1012.21	1.683	1.535	1.844	< .001
With	2	593.41	337.04	0	108.73	0.00	∞	-	-	.970
**Sarcoidosis**										
Without	761	57,340.90	1327.15	1263	124,877.32	1011.39	1.671	1.524	1.832	< .001
With	0	0.00	-	0	7.59	0.00	-	-	-	-
**HIV**										
Without	759	57,233.06	1326.16	1263	124,864.57	1011.50	1.670	1.523	1.830	< .001
With	2	107.85	1854.49	0	20.34	0.00	∞	-	-	.990
**Urbanization level**										
1 (The highest)	239	17,642.70	1354.67	460	42,581.05	1080.29	1.597	1.457	1.750	< .001
2	348	24,791.59	1403.70	608	57,101.20	1,064.78	1.679	1.532	1.840	< .001
3	30	3228.48	929.23	41	6408.10	639.82	1.849	1.687	2.027	< .001
4 (The lowest)	144	11,678.13	1,233.07	154	18,794.56	819.39	1.916	1.748	2.101	< .001
**Level of care**										
Hospital center	379	22,314.91	1698.42	694	55,941.34	1240.59	1.743	1.591	1.911	< .001
Regional hospital	316	25,700.37	1229.55	471	51,400.65	916.33	1.709	1.559	1.873	< .001
Local hospital	66	9325.62	707.73	98	17,542.91	558.63	1.613	1.472	1.768	< .001

HR = Hazard Ratio, CI = Confidence Interval, Adjusted HR: Adjusted variables listed in the table

*P*: Chi-square/Fisher’s exact test for categoricaly variables and t-test for continuous variables

CI: Confidence Interval

PYs: Person-years

Table 4 shows the factors of secondary lung cancer among different primary cancer types. The primary cancer types, including head and neck, colorectal, soft tissue sarcoma, breast, kidney, and thyroid cancer, have a significantly higher risk of developing secondary lung cancer in TB-infected patients. Nevertheless, bone, melanoma, and testicular cancer show no difference.

**Table 4:** Factors of secondary lung cancer among different primary cancer types using Cox regression and Fine & Gray’s competing risk model.

*TB*	With				Without *(Reference)*				Competing risk in the model			
Primary cancer types	Population	Events	PYs	Rate (per 10^5^ PYs)	Population	Events	PYs	Rate (per 10^5^ PYs)	Adjusted HR	95% CI	95% CI	*P*
**Overall**	6934	761	57,340.90	1327.15	13,868	1263	124,884.91	1011.33	1.671	1.525	1.832	< .001
Head and neck	2563	236	18,422.48	1281.04	5126	356	44,308.93	803.45	1.922	1.623	2.276	< .001
Colorectal	2560	312	21,330.60	1462.69	5120	571	45,984.63	1241.72	1.521	1.321	1.750	< .001
Bone	54	4	367.11	1089.59	108	14	1083.85	1291.70	1.366	0.397	4.703	.621
Soft tissue sarcoma	121	19	970.68	1957.40	242	28	1712.78	1634.77	2.104	1.103	4.013	.024
Melanoma	63	10	462.20	2163.58	126	21	1153.24	1820.96	1.081	0.475	2.464	.852
Breast	705	94	6728.86	1396.97	1410	131	13,787.25	950.15	2.058	1.569	2.700	< .001
Testicular	18	0	190.42	0.00	36	2	261.48	764.88	0.000	-	-	.963
Kidney	628	58	6306.78	919.65	1256	106	12,601.34	841.18	1.391	1.001	1.932	.049
Thyroid	222	28	2561.77	1092.99	444	34	3991.41	851.83	1.642	1.007	2.787	.046

HR = Hazard Ratio, CI = Confidence Interval, Adjusted HR: Adjusted variables listed in the Table

*P*: Chi-square/Fisher’s exact test for categorical variables and t-test for continuous variables

CI: Confidence Interval

PYs: Person-years

## Discussion

Previous studies conducted by the National Cancer Institute found the pulmonary TB was associated with an increased risk of lung cancer after adjusting for socioeconomic status and active smoking (odds ratio 2.1, 95% CI = 1.4–3.1) [5]. Epidemiological evidence concerning the association between pre-existing pulmonary TB and lung cancer has been documented [6–11]. Similarly, TB was associated with a 1.78-fold increase in lung cancer risk among nonsmokers and adenocarcinoma (relative risk: 1.6; 95% CI = 1.2–2.1) [4]. However, there is no large-scale study to discuss the association between TB-infected patients with subsequent metastatic cancer from other origins.

In our study, TB was associated with a 1.67-fold increase in the risk of secondary lung cancer compared with the non-TB cohort after adjusting for numerous potential confounders. The underlying mechanism of increasing cancer risk after TB infection had been reported. TB is thought to increase lung cancer risk through chronic pulmonary inflammation and fibrosis. TB infection may cause a profound and host immune response, with inflammatory cells in the lung producing extensive cytokine signaling cascades, oxygen species, reactive nitrogen, prostaglandins, and tissue-destructive proteases [12, 13]. The cell wall component of *Mycobacterium tuberculosis* can induce the production of nitric oxide and reactive oxygen species, which have been implicated in DNA damage leading to carcinogenesis [14].

It should be noted that nitrative DNA damage and oxidative DNA damage have been implicated in inflammation-related carcinogenesis [15]. Some data revealed that *Mycobacterium tuberculosis* might also enhance the synthesis of BCL-2, potentially leading to increased anti-apoptotic activity [16]. Chronic inflammation may also enhance lung fibrosis, which may be associated with decreased clearance of lymph and lymph-associated particles from the infected region [17]. Overall, the combination of DNA damage, anti-apoptosis, and the perpetuation of chronic inflammation may enhance progeny cell mutagenesis. These effects may lead to an increased risk of primary or secondary lung cancer.

We also found that all comorbidities increased the risk of secondary lung cancer significantly. This finding may be because more severe comorbidities were associated with the increased toxicity of specific treatments or the use of less aggressive or optimal treatment. These possibilities would thereby reduce the patient’s remaining life expectancy [18, 19]. In recent studies, the presence of comorbidities was significantly associated with elevated all-cause mortality in patients diagnosed with lung cancer, even after adjusting for sex, age, and cancer stage [20].

In our study, the study endpoint, the level of care, showed a significant difference. Compared with the non-TB cohort, TB-infected patients went to the local hospital (15.10% *vs* 11.85%; *p* < .001) and regional hospital (43.11% *vs* 38.43%) for treatments rather than to the medical center (41.79% *vs* 49.72%). This is because TB control and elimination relied on the early detection of active TB cases that prompted anti-TB treatment, identified persons at risk of exposure, and prevented secondary TB cases [21]. All of this depends on good diagnostic methods and effective treatments for TB. Thus, apart from medical care, the epidemiology of TB is increasing [22–24]. Outside of cities, most care is provided at the level of hospitals or lower at the local and regional levels. In these latter two instances, facilities may not be equipped to provide acute diagnoses and deliver effective treatment regimens. Thus, these patients’ characteristics are more at the lower urbanization level and go to their nearby local and regional hospital in the TB-infected study than non-TB patients. However, after adjusting for other risks (such as gender, age, insurance premium, related comorbidities, and urbanization level), the risk of secondary lung cancer was higher at the medical center (aHR = 2.332; 95% CI = 1.926–2.823; *p* < .001) and regional hospital (aHR = 1.728; 95% CI = 1.443–2.070; *p* < .001). The reason for this is that the NHI in Taiwan is a government-administered insurance-based national healthcare system. It is characterized by good accessibility, comprehensive population coverage, and relatively low costs [25].

Nevertheless, only the medical center and regional hospital having negative-pressure isolation wards that can isolate TB-infected patients. After they were discharged and developed secondary lung cancer, they went to their previous and familiar hospitals for help. This makes it a significantly high risk to “find” secondary lung cancer at medical centers and regional hospitals by 2.332- and 1.728 times than local hospitals, respectively.

After stratifying by variable factors using Cox regression and Fine & Gray’s competing risk model, we found that all factors increased the risk in TB-infected patients to develop lung cancer compared with non-infected patients. The Kaplan-Meier analysis revealed that TB-infected patients had a significantly higher risk of developing secondary lung cancer among primary cancer patients, even during the first year of tracking. The reasons for all the above phenomena are similar to those mentioned before. Also, these results demonstrated that TB-exposure is a risk for facilitating primary cancer to metastasize to the lung.

The primary cancer types, including head and neck, colorectal, soft tissue sarcoma, breast, kidney, and thyroid tumors, have a significantly higher risk of developing secondary lung cancer in TB-infected patients. Bone, melanoma, and testicular cancer show no difference. These results are similar to a previous study conducted on 228 cases with lung nodules. Most of the primary sites are colorectal in 25.8%, head and neck in 19.4%, urological organ in 14.7%, breast cancer in 10.5%, melanoma in 6.5%, and other primary sites (sarcoma, thyroid, squamous cell) in 6.1% [26]. Because the incidence of melanoma in Taiwan is relatively lower than that of Europe and America, the metastatic rate may not differ significantly. Also, bone and testicular tumors are more recurrent tumors and not distal metastases, so both did not show a difference.

Although this was a large-scale population-based nationwide study conducted from 2000 to 2015, it had some limitations. First, the patients’ ethnic background in this study was predominantly Asian, limiting the generalizability of these results. Second, the health insurance data we utilized did not include the histological stage and severity of primary cancer that may affect the metastatic ability. Third, our study also excluded laboratory results, such as sputum culture, exercise capacity, lifestyle data, nutrition supplements, and family history of systemic disease.

## Conclusions

In this study, TB was associated with a 1.67-fold increase in risk of secondary lung cancer compared with non-TB cohorts among the primary cancer. All comorbidities may increase the risk of developing secondary lung cancer. Therefore, clinicians should consider this in TB-infected patients, since TB leads to secondary lung cancer more easily among patients with primary cancer.

## Supporting information

S1 Table Abbreviation and ICD-9-CM codes. (DOCX)
