# Supplementary material for: Increased risk of secondary lung cancer in patients with tuberculosis: A nationwide, population-based cohort study
Source: PLoS One. 2021 May 7;16(5):e0250531. doi: 10.1371/journal.pone.0250531 (PMC8104424; doi:10.1371/journal.pone.0250531)
Supplement: S1 Table — (DOCX) [file pone.0250531.s001.docx]

**S1 Table. Abbreviation and ICD-9-CM codes**

|  | **Abbreviation** | **ICD-9-CM code** |
| --- | --- | --- |
| **Stduy populations:** Primary cancer |  |  |
| Head and neck |  | 140-149 |
| Colorectal |  | 153-154 |
| Bone |  | 170 |
| Soft tissue sarcoma |  | 171 |
| Melanoma |  | 172 |
| Breast |  | 174-175 |
| Testicular |  | 186 |
| Kidney |  | 189 |
| Thyroid |  | 193 |
| **Groups:** *Mycobacterium tuberculosis* | *TB* | 010-011 |
| **Events:** Secondary lung cancer |  | 197.0 |
| **Comorbidities:** |  |  |
| Diabetes mellitus | DM | 250 |
| Hypertension | HTN | 401-405 |
| Cirrhosis |  | 571.2, 571.5-571.6 |
| Congestive heart failure | CHF | 428 |
| Cerebrovascular acciden | CVA | 430-438 |
| Chronic kidney disease | CKD | 585 |
| Chronic obstructive pulmonary disease | COPD | 491-492, 496 |
